# Essentials of Laboratory Diagnosis

**Published:** 1910-11

**Authors:** 


**Essentials of Laboratory Diagnosis.** By **Francis Ashley Faught,** M.D., Director of the Laboratory of the Department of Clinical Medicine in the Medico-Chirurgical College of Philadelphia. Second edition. 12 mo, pp. 336. Illustrated. Philadelphia: F. A. Davis Co. 1910. (Cloth, $2.00.

This book is well suited to the wants of medical students and also to those physicians who maintain a small laboratory in connection with everyday work. As its title implies, it contains the essentials of those procedures necessary to clinical laboratory diagnosis and its peculiar value lies in the practical manner in which each subject is treated and each test described. In this second edition the author has brought the book well up to date. It should find a place in the laboratory of every practising physician. J. A. R.

